# Evaluation of antimicrobial susceptibility of *Escherichia coli *isolated from contaminated areas of Majengo slum in Meru County, Kenya

**DOI:** 10.12688/f1000research.124121.2

**Published:** 2023-01-26

**Authors:** Jared Ombuya, Kennedy Gachoka, Kagendo Dorothy, Naomi Mutea

**Affiliations:** 1 Meru University of Science and Technology, Meru, Kenya; 2 School of Applied Sciences, Meru University of Science & Technology, Meru, Kenya; 3 School of Health Sciences, Meru University of Science & Technology, Meru, Kenya; 4 School of Nursing, Meru University of Science and Technology, Meru, Kenya

**Keywords:** E. coli, Susceptibility Testing, Antimicrobial Resistance, Multidrug Resistance, Ciprofloxacin, Ceftazidime, Cefotaxime, Imepenem, Cefoxitin

## Abstract

**Background:** Antimicrobial drug resistance is of great concern today. Infections by the antimicrobial resistant strains of *Escherichia coli*, including enteropathogenic as well as enterotoxigenic strains have been reported as a major cause of deaths, especially among young children in low- and middle-income countries. This has been augmented by antimicrobial misuse, over the counter availability and poor sanitation especially in low income areas.

This study aimed at characterizing antimicrobial resistant strains of *Escherichia coli* isolated from sanitation environments of the Majengo slum in Meru County, Kenya

**Methods**: A cross-sectional study was conducted on 61 samples from soil, water and drains swabs. These were tested against five antimicrobial drugs by the Kirby disk diffusion method.

**Results:** A total of 42 (69%) of the samples had *Escherichia coli.*These recorded antimicrobial drug susceptibility as follows: Out of the five antimicrobial agents used, ceftazidime 28 (66.67%) showed the highest sensitivity followed by ciprofloxacin 26 (61.90%) and imepenem 25 (59.52%) respectively. cefotaxime and cefoxitin showed least sensitivity at 14 (33.33%) and 13 (30.95%) respectively. In intermediate imepenem and ciprofloxacin were the highest with 12 (28.57%) followed by cefotaxime 10 (23.81%). The least intermediate was observed in ceftazidime and cefoxitin both at 7 (16.67%). The highest resistance was observed in cefoxitin 22 (52.38%), followed by cefotaxime at 18 (42.86%). Ciprofloxacin, imepenem and ceftazidime had the lowest resistance 4 (9.52%), 5 (11.91%) and 7 (16.67%) respectively. The p-value <0.05 was considered significant to the study.

**Conclusions:** This study showed that *Escherichia coli* isolated from Majengo is pathogenic and resistant to antibiotics. Detection of *Escherichia coli* poses a great risk in the spread of resistant strains in human. Proper sanitation and hygiene awareness practices should be provided through education to the residents of this area.

## Introduction

Infectious organisms are currently the major cause of diseases worldwide. A number of newly recognized pathogens and strains are now emerging. These organisms have resulted in high morbidity and mortality globally. ^1^ The emergence of these pathogens has been attributed to microbial evolution, high mutations as the organisms try surviving in different environments as well as creation of new environments. This microbial evolution and high mutations has resulted to drug resistance, especially due to deposition of drug residues released in different environments, and more especially in areas with poor drainages and poor sanitation systems. As a result, there has evolved a need for surveillance and monitoring systems with emphasis on sanitation and water management. This will help curve the spread of emerging and re-emerging infectious diseases.

Human population growth currently has faced great challenges in accessing proper quality and quantity water resources. This has led to an increase in the number of water borne infectious diseases recently. ^2^ An estimated 30% of the bacterial population has been reported to be emerging because of wastewater, agricultural practices, and poor sanitation systems. Water management could act as a barrier to prevent the spread of pathogens. Prevention of pathogens could reduce environmental contamination, antibiotic misuse and eventually mutation of microorganisms.

In different environmental set ups, studies have indicated increased multidrug resistance strains which has led to increased mortality and morbidity resulting from exposure to infection causing organisms. Improved sanitation facilities is one of the most important interventions needed in order to stop the spread of resistant bacteria. ^3^ A study on typhoid among young children, ^4^ stressed the need for careful monitoring of antimicrobial resistance in a view to prevent increase of resistance strains and resultant infections to vulnerable communities.

Diarrheagenic *E. coli* has demonstrated a significant resistance to beta lactams antibiotics that are commonly prescribed, ^5^ with contributing factors to diarrhea cases attributed to poor quality of foods, poor water systems, lack of proper hygiene often due to lack of water and poor sanitation. Studies have shown that human and animal waste are the main sources of contamination on the environment that hosts strains that are antimicrobial resistance. ^6^ The study indicated that contamination resulted in formation of biofilms that supported bacterial resistance.

## Methods

### Ethical considerations

Approval to carry out the study was done by Meru University of Science and Technology MIRERC (Meru University Institutional Research and Ethics Review Committee). Approval Ref NO: MU/1/39/28 VOL.2(31). Date 17 ^th^ February 2022.

### Study area

The study was conducted in the Majengo area, an informal settlement in Meru County within the Eastern region of Kenya. As with other slum areas, Majengo has congested households with poor sanitation and limited access to safe toilet facilities. It is located at Imenti North sub-county and Ntima west ward. River Kathita passes along the slum.

### Study design and population

The study employed a cross-sectional study design with an aspect of laboratory analysis. Environmental samples were collected from water collection points, soils near latrines, and open drains in the Majengo area. Samples that were collected included water, soils, and swabs of open drains. The samples were then transported to the laboratory for analysis. The study did not involve households or interactions with residents of the Majengo area.

### Sampling procedure

**Figure 1.**
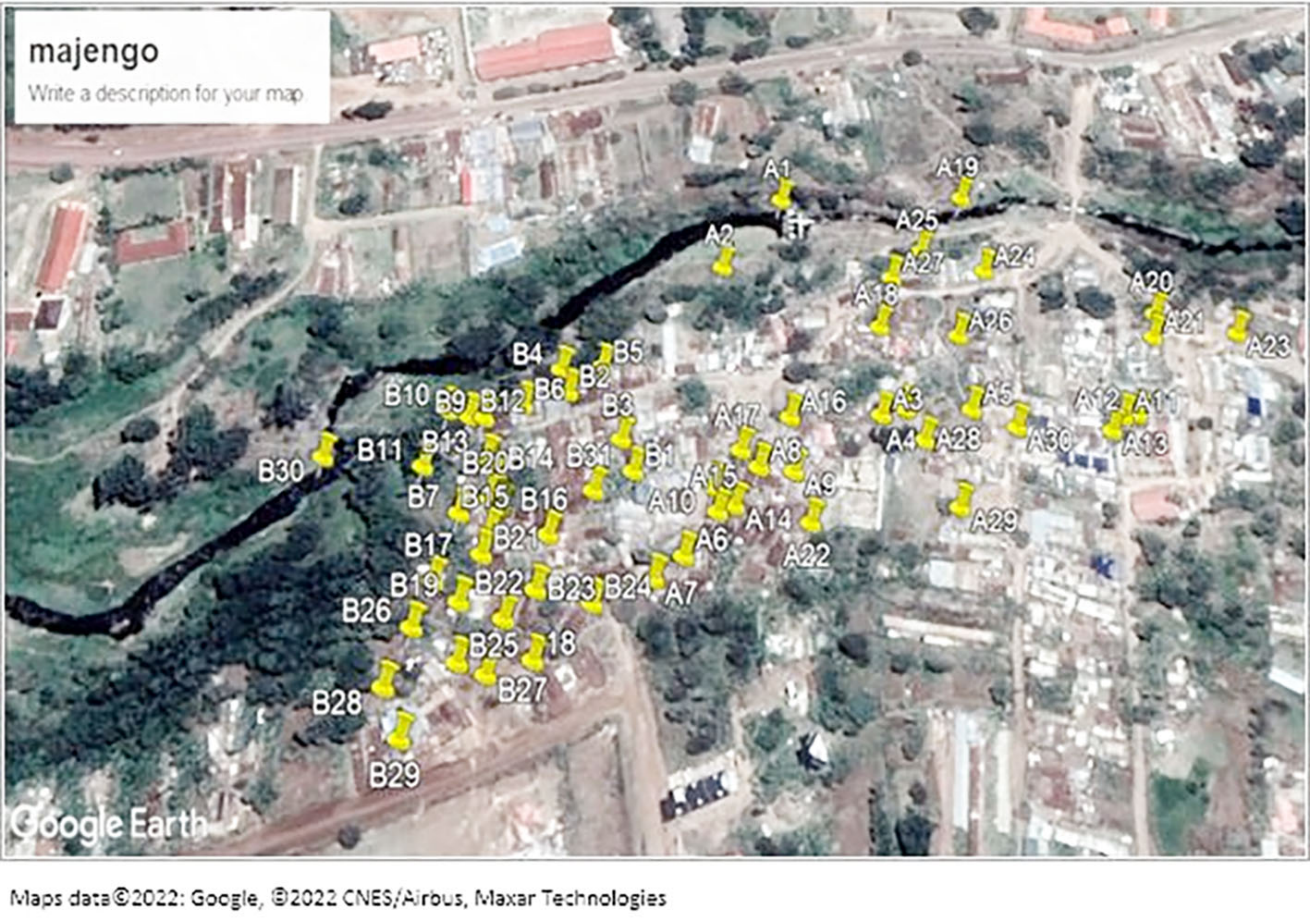
Shows a map of the sampled areas in the Majengo slum. Maps data ©2022: Google, ©2022 CNES/Airbus, Maxar Technologies.

To identify sites for sample collection, the study utilized a stratified sampling scheme, where the Majengo area was stratified by its constituent villages using available maps. In each village, areas of sampling were randomly selected based on availability of the water collection points, soils near latrines, and open drains. The number of sampled areas or type of samples in each village was dependent on accessibility and spatial/temporal distribution of the sample types. The sampled areas were marked using Google map to pin sample collection points. Figure 1 shows sampled areas in the Majengo slum. The area was divided into two strata A and B as shown on the map.

### Sample size determination

To determine the required sample size, the following formula was used at a 95% confidence interval. ^7^

$$n=\frac{{\left(z-\text{score}\right)}^{2}\times \text{StdDev}\times \left(1-\text{StdDev}\right)}{{\left(\text{marginal error}\right)}^{2}}$$

$$n=\frac{\left(1.96\right)2\times 0.2\left(1-0.2\right)}{0.05\times 0.05}$$

Sample size=61samples

### Sample processing

A 500 ml river water sample was collected into 500 ml sterile bottles by laying the bottle in water facing upstream. Tap water was collected into sterile 500 ml bottles. To do this, the individual tap was sterilized using a flamed alcohol cotton swab, a little water was allowed to flow, before getting collected into the sterile sample bottle. Approximately 10 g of soil samples were collected near toilets and dumping areas using a sterile spoon. In the swab collection, the cover was first untwisted to remove the swab and the sample collection was done by moving the swab in a clockwise circular motion. The swab was then returned aseptically into the vial containing transport media. It was labelled and packed in a cooler box containing an ice pack for transportation to the laboratory for analysis.

### Samples preparation prior to the inoculation

**Soil sample**

A sample of soil weighing 1g was dissolved in sterile distilled water in a 250 ml conical flask. ^8^ A serial dilution was performed up to *10 ^5^* for *E. coli* all dilutions were cultured into MacConkey agar (oxoid CM007) and incubated (Biobase) China at 37°C for 24 hours using microbiological standards of culturing. After this duration pink colonies of target organisms were sub cultured in Tryptone Bile Glucuronide (TBX) CM 0964 (Techno Pharmchem, India) to detect *E. coli.* Confirmation of *E. coli* was done on indole test.

**Drain swabs**

In the laboratory, drain swabs samples were cultured in MacConkey agar (Oxoid CM007) to isolate *E. coli.* The media was incubated at 37°C for 24 hours. After 24 hours’ growth pink colonies of *E. coli* in MacConkey agar (Oxoid CM007) were sub cultured in TBX to detect *E. coli.* Growth of green colonies was an indicator for the presence of *E. coli.*

### Water analysis using most probable number method

The most probable number method was used to analyze water samples using MacConkey broth (Oxoid CM0505CM 0505) UK. Each bottle with sample was appropriately labelled. The water was mixed thoroughly by inverting the bottle several times. The cap of the bottle was removed and the mouth of the bottle flamed. The water samples were then inoculated by arranging the bottles in an incubator independently inside the incubator grill. ^9^ After inoculation the bottles were incubated at 44°C for 24 hours and the samples were examined by observing the color change and gas formation. Table 1 shows water analysis set for untreated and treated water samples.

**Table 1.** Water analysis set for untreated and treated water samples.

Sample type	No. of bottles	Ml of broth	Strength of broth
Untreated water	1	50	Double
	5	10	Double
	5	5	Single
Treated water	1	50	Double
	5	10	Double

### Microbiological identification of *E. coli* from the samples

In the laboratory, samples were cultured in MacConkey agar to isolate *E. coli.* The cultures were incubated at 37°C for 24 hours. After 24 hours’ growth of target organisms in MacConkey agar were sub cultured in TBX to detect *E. coli.* Growth of green colonies was an indicator for the presence of *E. coli.*

### Biochemical identification of *E. coli* using Tryptone water

Isolates were subjected to biochemical identification using the indole test for *E. coli.* The test organism was inoculated in a bijou bottle containing 3 ml Tryptone water (Oxoid CM0087) UK and incubated at 37°C for 48 hours. The test for indole was done by adding one drop of Kovac’s reagent (Himedia) and the formation of a red ring within 10 minutes indicated presence of *E. coli.*

### Gram staining technique

Gram staining is a technique used in differentiation of gram-positive and gram-negative bacteria. The technique is important since it helps in selecting the correct antibiotic during microbial sensitivity. A smear was prepared from the colonies to confirm if it was gram-negative rods using gram staining procedure. The procedure required gram stain reagents, slides, oil immersion and a microscope.

Colonies of the test organism were emulsified on a glass slide using a wire loop. A drop of normal saline was added to make a smear. The slide was allowed to air dry. The slides were passed through a flame to fix them. The smear was covered with crystal violet solution for 30 seconds. Then the smear was rapidly washed off to remove the excess stain. The smear was covered with Gram’s Iodine (Pro-Lab Diagnostics Netherland) for 30 seconds and washed off using tap water. The smear was decolorized by covering with acetone for a few seconds and washed off. The smear was then covered with a counter stain neutral red for 30 seconds. The smear was allowed to dry before being examined at power ×100 on a microscope (Olympus CX22). Presence of gram-negative rods confirmed the organism.

### Antimicrobial susceptibility testing

Antimicrobial susceptibility testing was done using Mueller-Hinton Agar. Before inoculation of the organism to the Mueller-Hinton (CMO337 Oxoid) UK, single colonies were used to make 0.5 ml McFarland standard. ^10^^,^^11^ Colony suspension was first prepared by picking single colonies and suspending them in a vial containing 1ml normal saline. The suspension was compared with McFarland’s standard in order to obtain the required concentration of the isolate. Using a sterile cotton swab, the isolate was picked and spread evenly on Mueller-Hinton agar. Once all the Mueller-Hinton agar was completely inoculated by suspension, antimicrobial discs were inserted on the swabbed Mueller-Hinton agar. ^12^ The discs were well spaced to prevent them from overlapping. They were pressed on the media to ensure complete contact. They were incubated for 24 hours at 37°C. After 24 hours of incubation, the susceptibility and resistance was determined by measuring the diameter of the zone of inhibition using Vernier Calipers. The measurement was recorded in millimeters. This enabled classification of isolates of *E. coli* as either sensitive, intermediate, or resistant. ^11^^,^^13^ ATCC 25922 *E. coli* was used as a control organism during the study. The microbial agents used were as follows in their corresponding concentrations; imepenem (10 μg); ceftazidime (30 μg); cefotaxime (30 μg); cefoxitin (30 μg) and ciprofloxacin (5 μg).

### Data analysis

Laboratory data was entered into Microsoft Excel v 2010 and analyzed using SPSS version 26.0. Descriptive analysis was done using Microsoft Excel. The analysis included, frequencies and graphs. Data was compared between each study strata and between each sample type using Kruskal-Wallis tests, between the two drugs using the Wilcoxon Signed Rank test and findings were presented as figures tables and graphs. A p-value of 0.05 or less was considered significant between comparison in susceptibility and resistance between the strata A and B.

## Results

### Antimicrobial susceptibility from both strata

**Figure 2.**
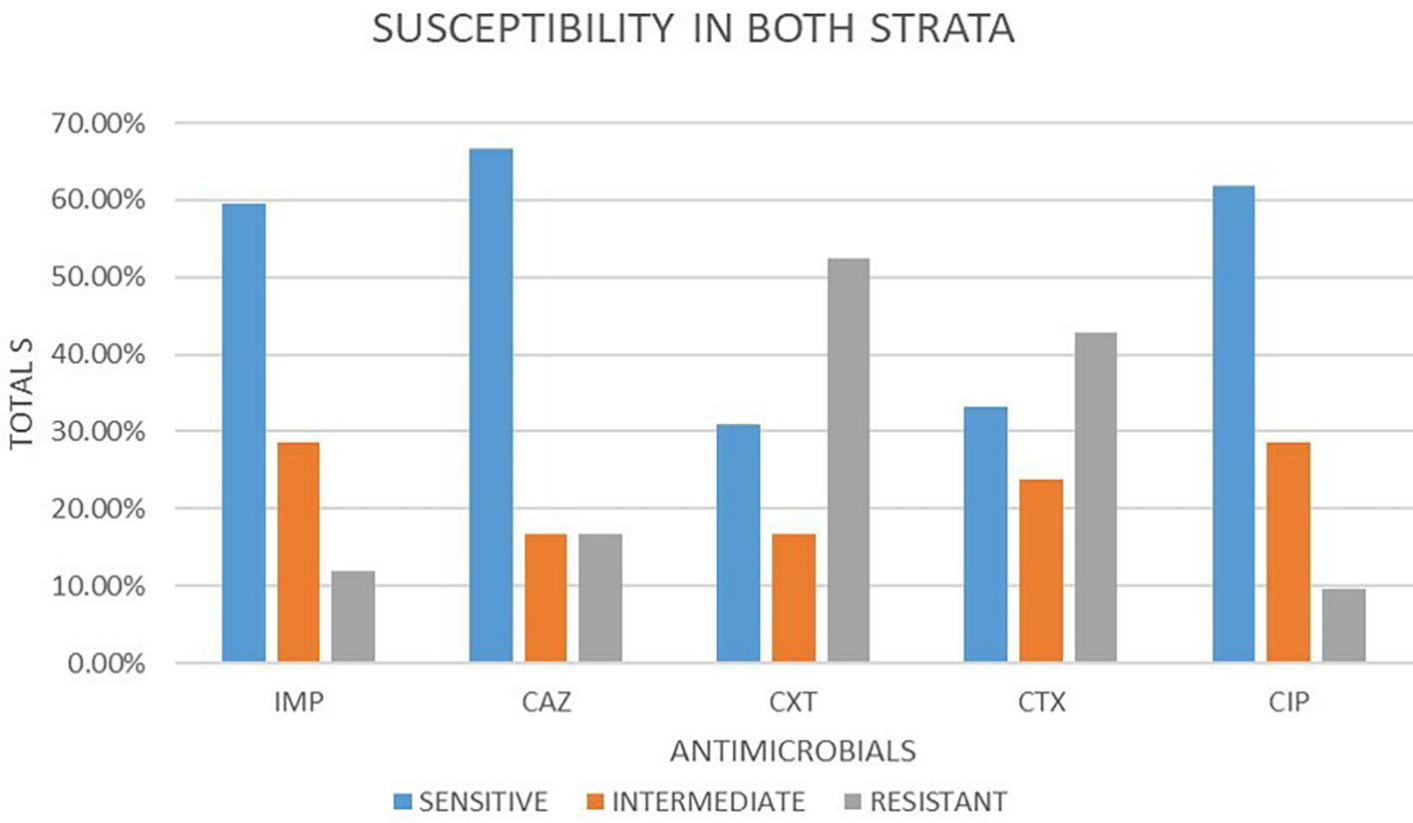
Shows a bar plot of the percentages of susceptibility patterns of different drugs under classes of response to *E. coli* in both strata. IMP-imipenem; CAZ-ceftazidime; CXT-cefoxitin; CTX-cefotaxime; CIP-ciprofloxacin.

A total of 42 isolates were tested for antimicrobial susceptibility in both strata. Out of these antimicrobials, 5 (50.48%) were susceptible, 5 (22.86%) were intermediate and 5 (26.67%) were resistant. Out of the five antimicrobial agents used, ceftazidime 28 (66.67%) showed the highest sensitivity followed by ciprofloxacin 26 (61.90%) and imepenem 25 (59.52%) respectively. cefotaxime and cefoxitin showed least sensitivity at 14 (33.33%) and 13 (30.95%) respectively. In intermediate imepenem and ciprofloxacin were the highest with 12 (28.57%) followed by cefotaxime 10 (23.81%). The least intermediate was observed in ceftazidime and cefoxitin both at 7 (16.67%). The highest resistance was observed in cefoxitin 22 (52.38%), followed by cefotaxime at 18 (42.86%). ciprofloxacin, imepenem and ceftazidime had the lowest resistance 4 (9.52%), 5 (11.91%) and 7 (16.67%) respectively. Figure 2 shows a bar plot of the percentages of susceptibility patterns of different drugs under classes of response to *E. coli.*

### Antimicrobial susceptibility from drain swabs

**Figure 3.**
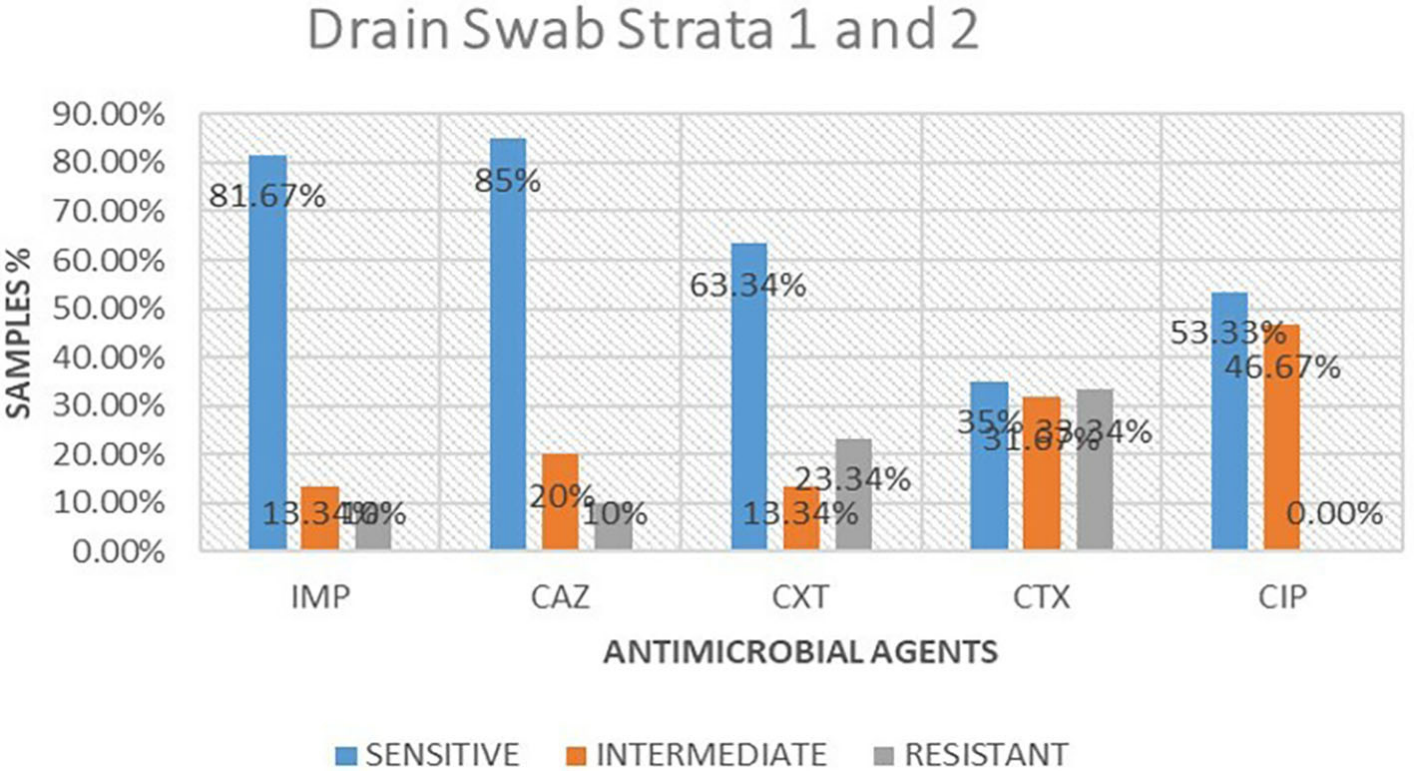
Shows antimicrobial susceptibility pattern from drain swabs in both the strata. IMP-imipenem; CAZ-ceftazidime; CXT-cefoxitin; CTX-cefotaxime; CIP-ciprofloxacin.

A total of 16 isolates from drain swabs were tested for antimicrobial susceptibility. Out of these antimicrobials, 5 (61.25%) were susceptible to the microbial, 5 (23.75%) were intermediate and 5 (15%) were resistant. The highest resistance was shown to be cefotaxime 6 (33.34%), followed by cefoxitin at 4 (23.34%), while imipenem and ceftazidime were the least resistant at 2 (10%) respectively. ciprofloxacin was 0% resistant to the isolates tested. Table 2 shows antimicrobial susceptibility patterns from drain swabs in both strata. Out of the five drugs ceftazidime showed the highest susceptibility at 13 (85%) while imipenem followed at 13 (81.67%) respectively. ciprofloxacin was the highest in intermediate at 8 (46.67%) while imipenem and cefoxitin were the least intermediate at 2 (13.34%). Figure 3 shows antimicrobial susceptibility pattern from drain swabs.

**Table 2.** Antimicrobial susceptibility frequencies from drain swabs in both strata.

Antimicrobial	Sensitive	Intermediate	Resistant
Imipenem	13(81.67%)	2(13.34%)	1(10%)
Ceftazidime	13(85%)	2(20%)	1(10%)
Cefoxitin	10(63.34%)	2(13.34%)	4(23.34%)
Cefotaxime	5(35%)	5(31.67%)	6(33.34%)
Ciprofloxacin	8(53.33%)	8(46.67%)	0(0.00%)

### Antimicrobial susceptibility from soil isolates from both strata

**Figure 4.**
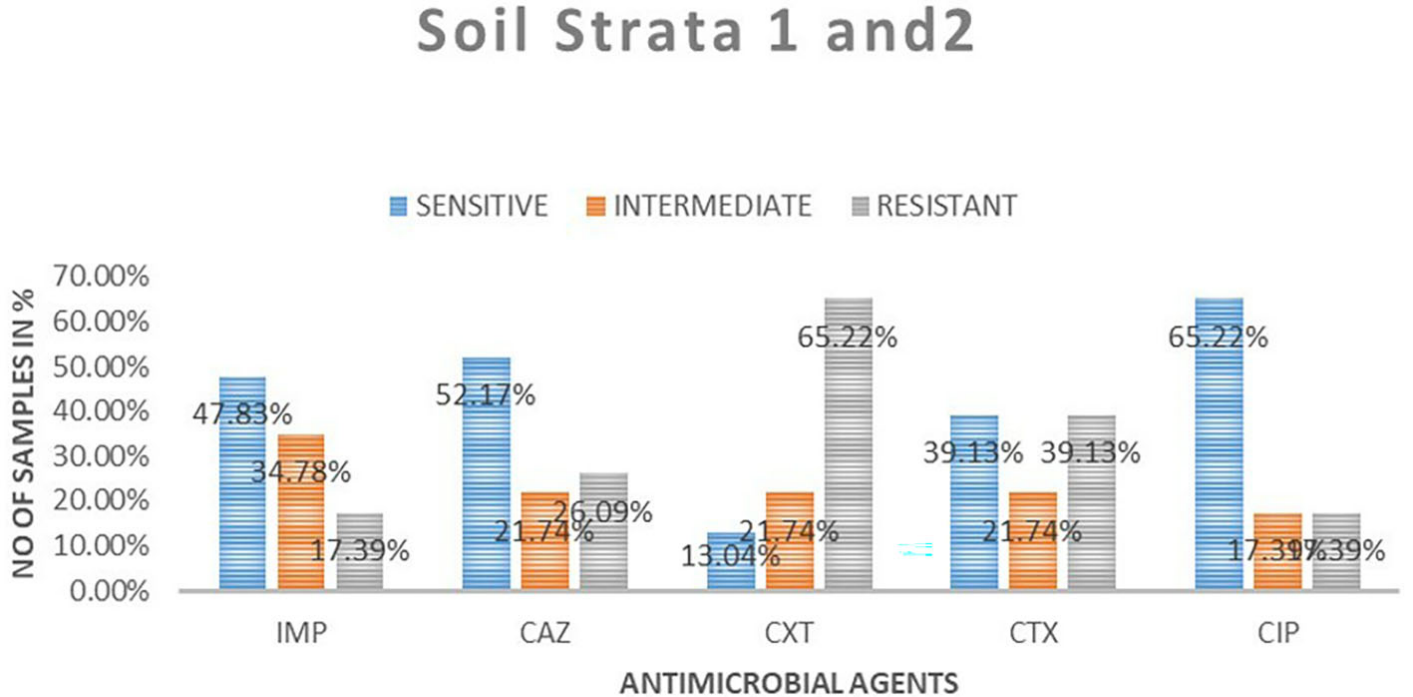
Shows the comparison in susceptibility patterns of the soil samples in both strata. IMP-imipenem; CAZ-ceftazidime; CXT-cefoxitin; CTX-cefotaxime; CIP-ciprofloxacin.

A total of 23 samples of soil were tested for antimicrobial sensitivity using diffusion disc method of the five antimicrobials of which imepenem resistant were 4 (17.39%), 8 (34.78%) intermediate while 11 (48.83%) susceptible. Out of the five antimicrobials used in this study in ceftazidime resistant were 6 (26.09%), intermediate 5 (21.74%), susceptibility 12 (52.17%), cefoxitin resistant were 15 (65.22%), intermediate 5 (21.71%), susceptible 3 (13.04%), cefotaxime resistant were 9 (9.13%), intermediate 5 (21.74%), susceptible 9 (39.13%) and ciprofloxacin resistant were 4 (17.39%), intermediate 4 (17.39%), susceptible 15 (65. 22%). Table 3 shows *E. coli* antimicrobials susceptibility drug patterns from soil samples in both strata. In both strata ciprofloxacin showed the highest susceptibility at 65.22%, followed by ceftazidime at 52.17%, while cefoxitin showed the least susceptibility *.* Imepenem showed the highest intermediate of all the drugs. Cefoxitin showed the highest resistance. Figure 4 shows the comparison in susceptibility patterns of the samples.

**Table 3.** *E. coli* antimicrobials susceptibility drug patterns from soil samples in both strata.

	Sensitive	Intermediate	Resistant	Total	Sensitive	Intermediate	Resistant
**Strata 1**							
Imipenem	7	2	0	9	77.78%	22.22%	0.00%
Ceftazidime	5	3	1	9	55.56%	33.33%	11.11%
Ceroxitin	2	1	6	9	22.22%	11.11%	66.67%
Cefotaxime	5	1	3	9	55.56%	11.11%	33.33%
Ciprofloxacin	6	2	1	9	66.67%	22.22%	11.11%
**Strata 2**							
Imipenem	4	6	4	14	28.57%	42.86%	28.57%
Ceftazidime	7	2	5	14	50.00%	14.29%	35.71%
Ceroxitin	1	4	9	14	7.14%	28.57%	64.29%
Cefotaxime	4	4	6	14	28.57%	28.57%	42.86%
Ciprofloxacin	9	2	3	14	64.29%	14.29%	21.43%

In the first strata, imepenem at 9 (77.78%) showed the highest susceptibility, followed by ciprofloxacin at 6 (66.67%) and cefoxitin was the least sensitive at 22.22%. Ceftazidime showed the highest intermediate at 33.33% while cefoxitin and cefotaxime were least intermediate. Cefoxitin showed the highest resistance while imepenem showed no resistant. In strata two ciprofloxacin 64.29% showed the highest susceptibility, followed by ceftazidime 50%. Cefoxitin showed highest resistance at 64.29%, followed by cefotaxime at 42.86%. Imepenem showed the highest intermediate of all the drugs.

### Antimicrobial susceptibility for water samples

**Figure 5.**
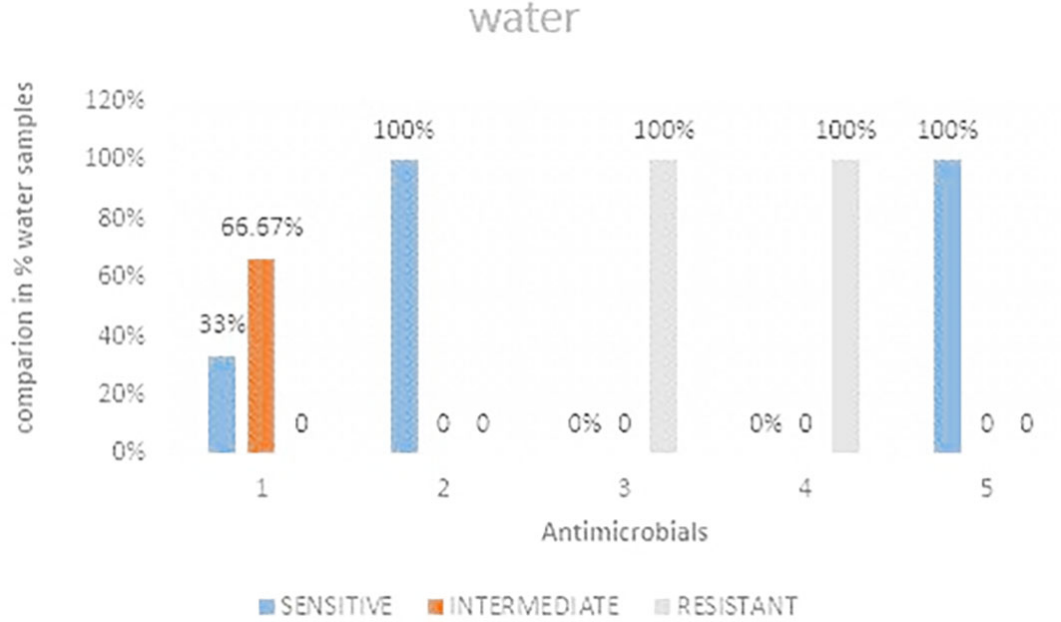
Shows the comparisons in percentages between the drugs for water samples. IMP-imipenem; CAZ-ceftazidime; CXT-cefoxitin; CTX-cefotaxime; CIP-ciprofloxacin.

Out of the three water samples analyzed it was observed that, ceftazidime and Ciprofloxacin showed 100% sensitivity while imipenem showed the least sensitivity Figure 5 shows the comparisons in % between the drugs. The highest resistance was observed in both cefoxitin and cefotaxime at 100%. Imipenem was the most intermediate at 66.67%. Table 4 shows *E. coli* antimicrobials susceptibility drug patterns from water samples from strata 1 and 2.

**Table 4.** *E. coli* antimicrobials susceptibility of water samples.

Water	Sensitive	Intermediate	Resistant	Total	Sensitive	Intermediate	Resistant
Imipenem	1	2	0	3	33%	66.67%	0%
Ceftazidime	3	0	0	3	100%	0%	0%
Cefoxitin	0	0	3	3	0%	0%	100%
Cefotaxime	0	0	3	3	0%	0%	100%
Ciprofloxacin	3	0	0	3	100%	0%	0

### Statistical comparison of resistance of the drugs using Kruskal-Wallis test and Wilcoxon Signed-Rank test

The resistance from this study was statistically significant since the p value=0.0005275354994284686 and the statistic=19.87938408896494. The p-value is less than 0.05, and so we reject the null hypothesis. This means that the resistance is not the same across the different drugs. To know which drug is more resistant, the Wilcoxon Signed-Rank test was used. This study found that cefoxitin and cefotaxime are statistically significant with a p-value 0.0156 which means they have highest resistance among the rest of the drugs. Table 5 shows p-values between compared drugs.

**Table 5.** p-values between compared drugs.

p-value	Ceftazidime	Cefoxitin	Cefotaxime	Ciprofloxacin
Imipenem	0.1088	0.0156	0.0156	0.7150
Ceftazidime		0.0156	0.0156	0.0679
Cefoxitin			0.2249	0.0156
Cefotaxime				0.0156

**Table 6.** Means and standard errors.

Drug	Mean±SE
Imipenem	23.809524±5.460448 ^a^
Ceftazidime	19.440476±9.395252 ^a^
Cefoxitin	9.333333±9.030211 ^b^
Cefotaxime	23.452381±4.695007 ^b^
Ciprofloxacin	21.547619±7.012888 ^a^

Means within a column followed by the same letter are not statistically different at p>0.05 as to resistance.

Imipenem, ceftazidime and ciprofloxacin are not significant to this study since the p value is greater than p>0.05. This p-value correlates to what was found in a study conducted by ^14^ who recorded a p-value of 0.36 towards *E. coli.*

**Figure 6.**
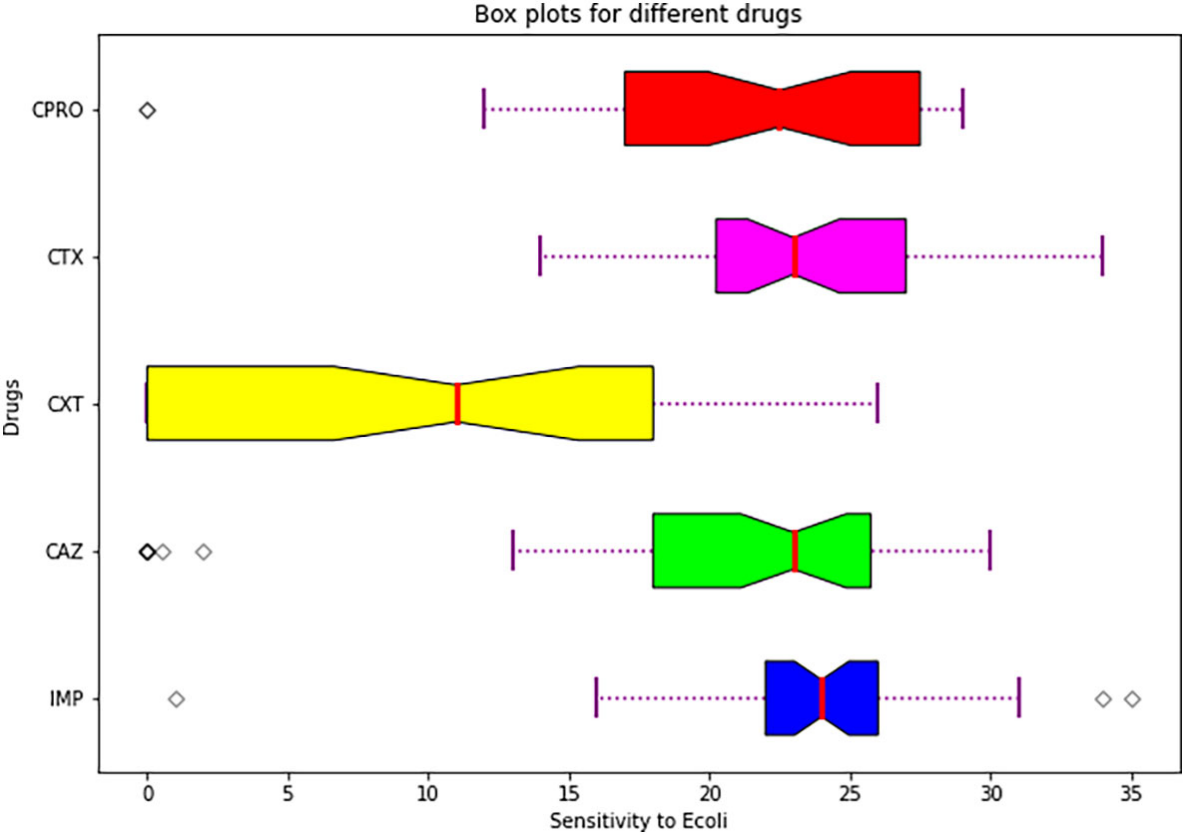
Shows a box plot showing a summary of descriptive statistics of the means and standard errors of the individual drugs used.

Statistical analysis summary using plot box. Figure 6 shows a box plot showing summary of descriptive statistics of the means and standard errors of the antimicrobials.

### Statistical comparison of sensitivity of the drugs using Kruskal-Wallis test and Wilcoxon Signed-Rank test

Sensitivity from this study was significant to the study since the p value=0.01171770477419787 between the strata. This means that the sensitivity was not the same across the different drugs. To know which drug is more sensitive, the Wilcoxon Signed-Rank test was performed. It was observed that, ciprofloxacin, ceftazidime and imipenem were the most sensitive drug with a p-value>0.05.

Table 7 shows the p-values of the antimicrobials in the study.

**Table 7.** p-values of the antimicrobials.

p-value	Ceftazidime	Cefoxitin	Cefotaxime	Ciprofloxcin
Imipenem	0.4630	0.0156	0.0277	0.9375
Ceftazidime		0.0156	0.0260	0.3454
Cefoxitin			0.9165	0.1158
Cefotaxime				0.0156

## Discussion

### Antimicrobial resistance patterns of *E. coli* from the study area

This study reported a range of multidrug resistance to *E. coli* from all the samples analyzed. Therefore, there is high chance that *E. coli* isolated from this study area emerges from the surrounding poor unhygienic conditions and inadequate sanitation facilities from the residents. This findings agrees with a study on contamination of street food in Burkina Faso that enteric organisms emerge from cross contamination. ^15^ The same observation was recorded by. ^16^ A study conducted on antimicrobial profile in Juja and Kibera found cefotaxime, ceftazidime and ciprofloxacin recorded a greater than 30% of antimicrobial resistance ^17^ this result is much lower than the one reported in the current study that recorded 25% increase. A study conducted by ^18^ observed that this increase in resistance is due to use in antibiotics in the treatment of diseases associated with poor unhygienic conditions. This study found the highest resistance on soil where it was it was noted that cefoxitin 65.22% an indication that soil was contaminated in this area. These findings correlate with study on urban informal settlement on antimicrobial resistance on the environment. It was noted from this study that less attention is being given on this contamination and pose a great risk to antimicrobial resistance. ^19^ Another study noted that soil is a hotspot carrier of resistant genes. ^20^

The current study recorded 34% towards cefotaxime, imepenem10% and zero resistance to Ciprofloxacin. In a similar study, ^21^ recorded 0% resistance to Ciprofloxacin and 1.68% to imipenem and 8.94% cefotaxime. This finding on cefotaxime and imepenem is much lower than the ones from the current study. However, both studies recorded 0% to ciprofloxacin. According to Ref. 22 multidrug resistance to *E. coli* was noted on cefotaxime at 79.7% resistance. A related study ^23^ recorded 93% resistance to *E. coli.* The current study recorded a lower figure of 42.86% resistance to cefotaxime.

The current study showed the least resistance towards ceftazidime (16.67%) which is much lower that the resistance obtained from ^24^ 100%.

### Antimicrobial resistance from water samples

The current study showed 100% resistance to water samples analyzed during the study. This resistance is a likely indication that there is high contamination of wastewater that is in return used for home use by the residents. The current study only focused on river water samples passing through the study area and not water samples from the source area. This calls for a study in order to determine if there is contamination. The findings from the current study was higher by 20% from a related study conducted by Ref. 25 on antidrug resistance on Indian rivers. A study done by Ref. 26 while investigating resistance from water found that ceftazidime and ciprofloxacin had almost the same resistance; 1.7% and 1.8% respectively. These findings are higher than the current study which recorded 0% resistance on the drugs. According to Ref. 27, *E. coli* in wastewater plants was 60% resistant towards ciprofloxacin, which was higher than the current study at 0% resistance.

## Conclusion

Antimicrobial resistance noted from this study will result in an increase of infections caused by treatment failure hence, high mortality rates.

### Recommendations

Proper sanitation and hygiene awareness practices should be provided through education to the residents of this area. In the future, molecular methods should be used to look in more detail at the resistance genotype of the isolate from the study.

## Author’s contribution

**JO:** Developed the concept, wrote the project proposal, collected the research data, analyzed the data, and wrote the thesis.

**KG:** Corrected the concept, provided necessary guidance, and corrections at the proposal writing, data analysis, and thesis writing.

**NM:** Corrected the concept, provided necessary guidance, and corrections at the proposal writing, data analysis, and thesis writing

## Data availability

### Underlying data

Figshare: EVALUATION OF ANTIMICROBIAL SUSCEPTIBILITY OF ESCHERICHIA COLI ISOLATED FROM CONTAMINATED AREAS OF MAJENGO SLUM IN MERU COUNTY, KENYA. https://doi.org/10.6084/m9.figshare.20325345. ^28^ This project contains the following underlying data:

• my data.xlsx (The data from this study represents data using measures of central tendency and the findings in percentages and bar graphs and statistical analysis involved using Kruskal Wallis and Wilcoxon from SPSS software.)

Data are available under the terms of the Creative Commons Attribution 4.0 International license (CC-BY 4.0).
