# Supplementary material for: Evaluation of antimicrobial susceptibility of Escherichia coli isolated from contaminated areas of Majengo slum in Meru County, Kenya
Source: F1000Res. 2025 Nov 13;11:1133. Originally published 2022 Oct 4. [Version 4] doi: 10.12688/f1000research.124121.4 (PMC12743212; doi:10.12688/f1000research.124121.4)
Supplement: Peer review history

# Reviewer report: Reviewer response for version 4

**DOI:** 10.5256/f1000research.189846.r438452

**Published:** 2026-01-02

**Authors:** Mulugeta Kibret

**Affiliations:** 1 Bahir Dar University, Bahir Dar, Ethiopia

**1. Summary**

 This study investigates the presence and antimicrobial susceptibility of *Escherichia coli i*n environmental samples collected from the Majengo informal settlement in Meru County, Kenya. A total of 61 samples were collected and analyzed using standard microbiological techniques, along with Kirby-Bauer disk diffusion testing against five antibiotics: imipenem, ceftazidime, cefotaxime, cefoxitin, and ciprofloxacin. The results reveal a high prevalence of *E. coli* in the environmental samples, with significant resistance observed against cefoxitin and cefotaxime. In contrast, lower levels of resistance were noted for ciprofloxacin, imipenem, and ceftazidime. Statistical tests were applied to examine the resistance patterns among the antibiotics. The study concludes that inadequate sanitation likely serves as a reservoir for antimicrobial-resistant *E. coli, p*osing public health risks in informal settlements. It emphasizes the urgent need for improved sanitation, hygiene education, and ongoing surveillance of antimicrobial resistance.

**2. Evaluation of specific review questions**

**2.1 Is the work clearly and accurately presented and does it cite the current literature?**

**Yes,**

**Expanded justification:**

 The manuscript is well-structured, logically organized, and clearly written. The objectives are explicitly stated and aligned with the background. The authors reference a significant amount of recent literature, mostly from the last 5 to 7 years, effectively situating the study within the One Health framework and the context of environmental antimicrobial resistance. The discussion effectively compares the findings with similar studies conducted in Africa and other low- to middle-income settings, which enhances the external relevance of the results.

**Minor suggestions**

 The introduction could better align with global environmental AMR surveillance frameworks. Some stylistic edits could enhance clarity without impacting scientific accuracy.

**2.2 Is the study design appropriate, and is the work technically sound?**

**Answer: Yes**

**Expanded justification:**

 A cross-sectional environmental surveillance design is effective for characterizing antimicrobial susceptibility patterns in high-risk sanitation settings. By sampling across different environmental matrices, the study can capture a variety of exposure pathways. Standard laboratory methods are validated and suitable for assessing phenotypic AMR in resource-limited contexts.

**Minor limitations**

 The antibiotic panel is limited to five agents, primarily β-lactams. Broader coverage with tetracyclines and aminoglycosides could improve future studies. 

 Molecular characterization of resistance genes was not conducted, but it is recognized as a future research priority.

 These limitations do **not** undermine the technical validity of the current work.

**2.3 Are sufficient details of methods and analysis provided to allow replication by others?**

 Answer: Yes

 It specifies reagents, incubation conditions, antibiotic concentrations, and analytical software, providing enough detail for independent replication by other researchers in similar settings.

**2.4 If applicable, is the statistical analysis and its interpretation appropriate?**

**Answer: Yes**

 Expanded justification: The authors appropriately employ non-parametric tests, specifically the Kruskal-Wallis and Wilcoxon signed-rank tests, considering the sample size and data distribution. The statistical results are clearly presented, and interpretations are cautious and consistent with the descriptive findings. Although some analyses, such as antibiotic comparisons, are not crucial for the main conclusions, they are presented transparently and without any misleading interpretations.

**2.5 Are all the source data underlying the results available to ensure full reproducibility?**

**Answer: Yes**

**Expanded justification:**

 All data are publicly available on Figshare with documentation and open licensing, ensuring transparency, reproducibility, and allowing for reanalysis by other researchers.

**2.6 Are the conclusions drawn adequately supported by the results?**

 Answer: Yes

 The conclusions align with the results, focusing on phenotypic resistance and environmental contamination. The authors avoid overgeneralization and frame their findings within the study's limitations. The public health implications related to sanitation, hygiene, and AMR surveillance are reasonable and data-supported.

**3. Minor note:**

- Statements regarding pathogenicity are better interpreted in the context of environmental and public health risk rather than clinical virulence; the revised version has improved clarity in this regard.

**4. Overall assessment**

 This manuscript offers valuable evidence on environmental antimicrobial resistance in informal settlements and significantly contributes to the One Health AMR literature. The study design, methods, analysis, and conclusions are robust and align well with the objectives. With minor optional refinements, the article meets the standards for scientific rigor and reproducibility for indexing.

Is the work clearly and accurately presented and does it cite the current literature?

Yes

If applicable, is the statistical analysis and its interpretation appropriate?

Yes

Are all the source data underlying the results available to ensure full reproducibility?

Yes

Is the study design appropriate and is the work technically sound?

Yes

Are the conclusions drawn adequately supported by the results?

Yes

Are sufficient details of methods and analysis provided to allow replication by others?

Yes

Reviewer Expertise:

My area of expertise is microbiology and environmental AMR.

I confirm that I have read this submission and believe that I have an appropriate level of expertise to confirm that it is of an acceptable scientific standard.

# Reviewer report: Reviewer response for version 4

**DOI:** 10.5256/f1000research.189846.r438460

**Published:** 2025-12-30

**Authors:** K.B.M Saiful Islam

**Affiliations:** 1 Sher-e-Bangla Agricultural University, Dhaka, Bangladesh

This study investigates antimicrobial susceptibility patterns of *Escherichia coli* isolated from environmental samples collected in the Majengo slum of Meru County, Kenya. The topic is highly relevant given the global concern over antimicrobial resistance (AMR) in low‑resource urban settings. The authors employed a cross‑sectional design, collecting 61 environmental samples (soil, water, and drain swabs) and testing 42 confirmed *E. coli* isolates against five antibiotics using the disc diffusion method.

 The study addresses an important One Health issue; environmental AMR in informal settlements, and contributes surveillance data from an underserved Kenyan region. Version 4 incorporates several meaningful revisions, including sample size justification using Cochran’s formula, an extended conclusion linking findings to broader public health implications, and improvements to tables and figures. These revisions demonstrate responsiveness to earlier reviewer critiques, particularly regarding statistical misapplications highlighted by Reviewers 2 and 3.

 Despite these improvements, several issues remain that must be addressed to meet scientific standards:

**1. Literature Context**

 The introduction would benefit from deeper engagement with current AMR surveillance frameworks and comparative studies from similar slum environments. Incorporating recent WHO, FAO, and WOAH reports, as well as regional AMR data from East Africa, would strengthen the scientific grounding and contextual relevance.

**2. Study Design and Methods**

 Although the sampling strategy is described, the rationale for stratification and site selection requires clearer justification. The antimicrobial panel is appropriate; however, the study lacks molecular confirmation of resistance determinants, which limits interpretation of resistance mechanisms. While ATCC 25922 was used as a control strain, inclusion of additional quality‑control measures would enhance methodological robustness. Minor typographical errors (e.g., “imepenem” instead of “imipenem,” “ceroxitin” instead of “cefoxitin”) should also be corrected.

**3. Statistical Analysis**

 The use of Kruskal-Wallis and Wilcoxon Signed‑Rank tests is appropriate for non‑parametric data; however, the statistical reporting is minimal. P‑values are presented without effect sizes, confidence intervals, or justification for test selection. Interpretation of statistical significance should be linked to environmental variables, sample types, and potential confounders.

**4. Data Availability and Reproducibility**

 The manuscript does not provide raw data such as zone diameters, isolate‑level susceptibility profiles, or precise sampling coordinates. For full reproducibility, the authors should upload complete datasets and laboratory protocols to a public repository, as required by F1000Research’s open‑data policy.

**5. Conclusions**

 The conclusions regarding resistance patterns and public health risks are generally supported by the results. However, the statement that *E. coli* isolates are “pathogenic” is not substantiated, as no virulence profiling was performed. Recommendations on sanitation and hygiene are appropriate but should be framed within the study’s methodological limitations.

**Recommendations for Improvement**

- Expand the literature review with current AMR surveillance references and regional comparative studies.
- Provide clearer justification for sampling design, stratification, and site selection.
- Include raw data (zone diameters, isolate metadata) and upload full datasets to a public repository.
- Report effect sizes and confidence intervals alongside p‑values.
- Clarify study limitations and avoid overgeneralization regarding pathogenicity.

This study has the potential to contribute meaningfully to environmental AMR surveillance in informal settlements. Addressing the points above will substantially improve the scientific rigor, reproducibility, and interpretability of the work.

Is the work clearly and accurately presented and does it cite the current literature?

Partly

If applicable, is the statistical analysis and its interpretation appropriate?

Partly

Are all the source data underlying the results available to ensure full reproducibility?

Partly

Is the study design appropriate and is the work technically sound?

Partly

Are the conclusions drawn adequately supported by the results?

Partly

Are sufficient details of methods and analysis provided to allow replication by others?

Partly

Reviewer Expertise:

Antimicrobial Resistance, Microbiology, Veterinary Public Health, One Health, Environmental Surveillance, Zoonoses, Food Safety, Veterinary Medicine

I confirm that I have read this submission and believe that I have an appropriate level of expertise to confirm that it is of an acceptable scientific standard, however I have significant reservations, as outlined above.

# Reviewer report: Reviewer response for version 4

**DOI:** 10.5256/f1000research.189846.r439717

**Published:** 2025-12-26

**Authors:** Chimdi Mang Kalu

**Affiliations:** 1 University of South Africa, Florida, South Africa

The manuscript entitled "Evaluation of antimicrobial susceptibility of *Escherichia coli* isolated from contaminated areas of Majengo slum in Meru County, Kenya" presents the prevalence of antibiotic-resistant *E. coli* from soil, water, and drain swabs in a poor sanitation area in Kenya. The topic is interesting and relevant as it addresses the current issues of antimicrobial resistance of the bacterium that is known to cause a series of deaths. However, the manuscript needs some revisions as stipulated below:

**Abstract:**

*Escherichia coli* should be written once and subsequently be written as *E. coli*. In the method section, there was no information on how the 61 samples were obtained in total. Was the sampling done in triplicate or what?

 Then, in the results section, I recommend that it be rewritten. For example,

 “Out of the five antimicrobial agents used, ceftazidime 28 (66.67%) showed the highest sensitivity followed by ciprofloxacin 26 (61.90%) and imepenem 25 (59.52%) respectively.”

 “Ceftazidime does not show sensitivity; rather, 28 (66.67%) of isolated *E. coli* showed sensitivity to ceftazidime.” Please consider restructuring this section.

**Introduction:**

 This section provided general information on infectious diseases without adequate concentration on *E. coli* and the diseases or deaths caused.  There was no linked information on the occurrence of this bacterium in Kenya, making a clear justification of this study difficult. Hence, I recommend that the introduction be completely restructured with clear justification and objectives.

**Methods:**

 The ethical consideration statement should come at the end.

 In the sampling procedure, there was no mention of the number of samples collected from soil, water, and swab. Was the sampling done in duplicates or triplicates?

 What is the justification for using the size determination method?  How did you calculate the standard deviation and the Z-score value?

 “A serial dilution was performed up to 10 ^5^ for *E. coli* all dilutions were cultured into MacConkey agar (oxoid CM007) and incubated (Biobase) China at 37°C for 24 hours using microbiological standards of culturing.”  Consider splitting this sentence into two……..

 “Confirmation of *E. coli* was done on indole test.” Please indicate the expected results of this confirmation test……..

 Five antibiotics were used. However, there was no information on the classes of antibiotics to which they belong. This will help to identify the isolates that were multidrug resistant.

**Results:**

 This section needs to be rewritten. There is no need for intermediate resistance. It is either susceptible or resistant.

 “Out of the five antimicrobial agents used, ceftazidime 28 (66.67%) showed the highest sensitivity followed by ciprofloxacin 26 (61.90%) and imepenem 25 (59.52%) respectively.”

 “Ceftazidime does not show sensitivity; rather, 28 (66.67%) of isolated *E. coli* showed sensitivity to ceftazidime.” Please consider restructuring this section. Effect this throughout this section…………..

 “A total of 16 isolates from drain swabs were tested for antimicrobial susceptibility. Out of these antimicrobials, 5 (61.25%) were susceptible to the microbial,”…………………..Are you dealing with microbial or specifically *E. coli*? Please be specific…..

**Discussion:**

 The discussion should be strengthened to provide a clear impact of the study in unveiling the antimicrobial resistance of *E. coli* in Kenya.

 Conclusion:

 “This study confirmed the presence of Escherichia coli O157”

 How did you arrive at this strain? Was there any sequencing of the isolate done to give clear indication of the O157 strain?

 You can just write…..This study confirmed the presence of Escherichia coli

Is the work clearly and accurately presented and does it cite the current literature?

Partly

If applicable, is the statistical analysis and its interpretation appropriate?

Partly

Are all the source data underlying the results available to ensure full reproducibility?

Partly

Is the study design appropriate and is the work technically sound?

Partly

Are the conclusions drawn adequately supported by the results?

Yes

Are sufficient details of methods and analysis provided to allow replication by others?

Partly

Reviewer Expertise:

Molecular biology,  antibiotic resistance, virulence, and water analysis

I confirm that I have read this submission and believe that I have an appropriate level of expertise to confirm that it is of an acceptable scientific standard, however I have significant reservations, as outlined above.

# Reviewer report: Reviewer response for version 3

**DOI:** 10.5256/f1000research.144571.r176272

**Published:** 2024-09-12

**Authors:** Abdennaceur Hassen

**Affiliations:** 1 Research Center of Wastewater technologies, Technopark Borj Cedria, Soliman, Tunisia

**Main comments**

 As a whole, I find the article very interesting since it discusses a relevant topic, bacterial multidrug resistance, in a developed African country like Kenya, and a specific situation and environment. The study aimed to determine and provide crucial insights characterizing antimicrobial-resistant strains of *Escherichia coli* isolated from sanitation environments in the Majengo slum in Meru County, Kenya.

 This statement is clear and concise. This subject is of considerable relevance in light of the importance of infections by antimicrobial-resistant *Escherichia coli* strains, such as enteropathogenic and enterotoxigenic strains. These strains of *Escherichia coli* have been reported worldwide as a major cause of death, especially among young children in low- and middle-income countries, like most African countries. This major cause of death has been exacerbated by antimicrobial misuse, over-the-counter availability, and poor sanitation, especially in low-income areas.

 Additionally, I always recommend adding a graphical abstract to aid in understanding the study. The graphical abstract, sometimes not required and requested by the journal, gives added value. This helps the reader better understand the reality of the work.  

 On the other hand, the quality and presentation of the graphical abstract, in my opinion, is an excellent indicator concerning the research team. This is because it indicates the validity of their work, its seriousness, and its scientific rigor.

 The paper is relatively short. It will be a relatively short paper. The manuscript is well-written in English and sometimes requires corrections and intervention. It would be helpful if you could provide me with a Doc manuscript so that I can assist the authors.

 I summarize my main comments about this work:

**Abstract**

 I find the abstract well-written, expressive, well-structured, and well-extended. I recommend removing Multidrug resistance since it has the same meaning as “antimicrobial resistance”, and adding “E. Isolates" instead of just “E. coli” and “sanitation environments”. These two keywords should be more indicative and precise concerning this investigation.

**Introduction**

 A clear introduction to a topic that takes less than a page to introduce and explain the subject. It is nicely presented and properly defended which makes the topic and investigation useful. Furthermore, this introduction demonstrated the high relevance of the topic. I recommend some extension of this introduction considering the importance and abundance of the bibliography on this specific theme in the scientific literature. Of nearly all references cited, 27 references were not within the standards recommended by scientific journals of around 40 references; and once there is a small extension in the introduction, the number of references automatically increases a little. These references were almost all very recent since around 85% of the references were from 2021.

 After the introduction, the main goals of this study are thoroughly introduced, well-cited, and structured. These goals are clearly expressed and written.

**Materials and methods**

 All methodologies were well documented and described with all necessary monitoring, including microbiological and statistical tests. Although some corrections are needed to confirm the seriousness of the investigation. Regarding the sampling modality, it requires more explanation in terms of text restructuring. Gram staining technique description is not necessary since this technique is well-known and common.

**Results and discussion**

 Results queues and text citations are useful and consistent. All results are well-cited, clearly explained, and defended. Concerning this defense, I noticed that the authors cited some very recent references beyond 2021 when defending their results.

**Figures and tables**

 Although the illustrations are clear, clearly captioned, and well-made; however, Table 6 needs additional captions to aid in understanding and signifying the obtained values, ...etc.

**Conclusion**

 As a conclusion, I think it is too short, so I think it needs some extension.

**Decision**

 The work is well done, and consistent and deserves publication in a future issue with a correction. I mean English writing mainly. It is perfectly within the journal's scope.

 Last, and for all the reasons mentioned above, I recommend the indexing of this work with a minor correction.****

Is the work clearly and accurately presented and does it cite the current literature?

Yes

If applicable, is the statistical analysis and its interpretation appropriate?

Yes

Are all the source data underlying the results available to ensure full reproducibility?

Yes

Is the study design appropriate and is the work technically sound?

Yes

Are the conclusions drawn adequately supported by the results?

Yes

Are sufficient details of methods and analysis provided to allow replication by others?

Yes

Reviewer Expertise:

Économie de l'eau, Hydrologie environnementale, Microbiologie,  Antibiorésistance, Écologie environnementale et moléculaire, Écotoxicologie  Processus de traitement de l’eau et de Filtration.  Certification, normalisation  et accréditation.

I confirm that I have read this submission and believe that I have an appropriate level of expertise to confirm that it is of an acceptable scientific standard, however I have significant reservations, as outlined above.

# Response

**Published:** 2024-09-13

**Authors:** jared ombuya

**Affiliations:** meru university, Kenya

Thanks you for your comments I will do the corrections posted and share the updated version

 regards 

 Jared

# Response

**Published:** 2024-11-24

**Authors:** jared ombuya

**Affiliations:** meru university, Kenya

Will do the correction stated.

 Regards

# Response

**Published:** 2025-10-16

**Authors:** jared ombuya

**Affiliations:** meru university, Kenya

Graphical presentations were already added. Corrections have been done and a new version uploaded

# Reviewer report: Reviewer response for version 3

**DOI:** 10.5256/f1000research.144571.r176275

**Published:** 2023-06-19

**Authors:** Md. Abdus Sobur

**Affiliations:** 1 Microbiology and Hygiene, Bangladesh Agricultural University, Mymensingh, Mymensingh Division, Bangladesh; 2 Poultry Diagnostic Lab, Provita Group, Mymensingh, Mymensingh Division, Bangladesh

The manuscript entitled "Evaluation of antimicrobial susceptibility of Escherichia coli isolated from contaminated areas of Majengo slum in Meru County, Kenya" has presented the prevalence of antibiotic resistance E. coli from soil, water and drains swabs in a poor sanitation area in Kenya. The topic has been chosen by the Authors has importance in One Health view point. However, the study design has some serous faults particularly, in statistical analysis. In addition, the whole manuscript requires major revisions in terms of scientific writing and sentence corrections. Over all the manuscript is not at the stage of indexing. Due to no line number, line wise correction is not possible. I have written the report based in section.

 Abstract:

 The Authors can write as E. coli after first use. The result section should be rewritten. The authors have mentioned the sensitive prevalence first, and then intermediate finally, the resistance pattern. They should write the resistance pattern first and then sensitive pattern if they feel sensitive pattern is important. No need to mention the intermediate pattern in abstract. To me, only resistance pattern is enough for abstract section. In conclusion, there is no indication in results and methods of abstract to say E. col as pathogenic and even no clue in the whole manuscript. What is the basis of this finding?

 Keywords:

 Why did the Authors use multidrug resistance as keywords when they have not identified multidrug resistance in their study?

 Introduction:

 As the background and justification of the study "introduction" section is not well written. The Authors have studied on E. coli in a particular area and they have mentioned the area as "contaminated" in the title. However, they have not indicated any linkage to be the area as contaminated. In addition, they have not described the area, people live there and their occupation which are important for E. coli contamination as well as antimicrobial resistance (AMR) relation. This information would help to formulate the justification of the study. It was mainly to convince the reader why the area is contaminated with AMR E. coli.  Unfortunately, the justification of the study is absent here. Furthermore, there is no aim mentioned as well.

 Methods:

 Sample size determination:

 Seems the reference (7) is not correct. On top of that, the Authors have not mentioned the meaning of the values they used in the formula. In addition, as per the equation no. of samples will be 246 not 61. The calculation is correct.

 Sample processing:

 How much tap water was collected? The Authors collected 500 ml river water in 500 ml bottle that is not a good practice. The bottle should be larger than the collection amount to prevent contamination.

 Samples preparation prior to the inoculation:

 Although the Authors are saying sample preparation prior to inoculation however, they have mentioned the culture procedures here as well. And they have written the microbial techniques separately bellow. No need to write separately as already mentioned. It was best to identify the E. coli by molecular technique.

 Antimicrobial susceptibility testing:

 Five antibiotics were selected among them, three were cephalosporin, one carbapenem (these four are B-lactams) and one Fluoroquinolones. Therefore, categorically only two types of antibiotics was selected. For this selection, it was best to identify ESBL among the E. coli isolates.

 Data analysis:

 Before doing any statistical analysis, maintaining minimum sample number (from population) should be followed otherwise statistical analysis get lower importance. In addition, comparing among antibiotics in this study is not useful as most of the antibiotics fall in the same group. Comparing among group will not be useful as well as one B-lactams have four antibiotics and Fluoroquinolones has only one antibiotics. Therefore, this analysis does not add any value to this study.

Is the work clearly and accurately presented and does it cite the current literature?

No

If applicable, is the statistical analysis and its interpretation appropriate?

No

Are all the source data underlying the results available to ensure full reproducibility?

No source data required

Is the study design appropriate and is the work technically sound?

No

Are the conclusions drawn adequately supported by the results?

No

Are sufficient details of methods and analysis provided to allow replication by others?

Partly

Reviewer Expertise:

Antibiotic Resistance, Virulence, Veterinary Microbiology, Food Microbiology, Poultry Health, One Health

I confirm that I have read this submission and believe that I have an appropriate level of expertise to state that I do not consider it to be of an acceptable scientific standard, for reasons outlined above.

# Reviewer report: Reviewer response for version 3

**DOI:** 10.5256/f1000research.144571.r176271

**Published:** 2023-06-13

**Authors:** Benno H Ter Kuile

**Affiliations:** 1 Swammerdam Institute of Life Sciences, University of Amsterdam, Amsterdam, North Holland, The Netherlands

This manuscript describes the level of resistance of *Escherichia* *coli* isolated from soil, water and drains swabs in a poor sanitation area in Kenya. The data are of interest as information about antimicrobial resistance in such environment is essential for a global OneHealth approach. While the data collection has been performed correctly, the data processing is substandard. A considerable revision of the results section will be needed.

 The application of statistics is incorrect in many instances, but luckily often not required. I have looked up reference 7 on the sample size, but I could not find the equation the authors use. However, the final sample size (41 E. coli positive samples) is on the low side, but that does not invalidate the study. Tables 5 and 7 and figure 6 are useless, because antibiotics are not experimental groups, so the p-values are meaningless.

 Throughout the study numbers are mentioned that are either incomprehensible, or don’t add up. In table 2, first two lines, the same numbers do not have the same percentages and they don’t add up to 100. Line 3: if 10 = 63.34%, then 2 cannot be 13.34 and if 2 is 13.34, then 4 cannot be 23.34. This is only one example, but throughout the manuscript numbers are flawed, don’t add up and simply don’t make sense.

 A sentence like “Out of these antimicrobials, 5 (50.48%) were susceptible, 5 (22.86%) were intermediate and 5 (26.67%) were resistant.” is impossible to interpret. Antimicrobials are not susceptible, intermediate or resistant; microbes are or are not.

 One final example: throughout the text the need for monitoring is stressed as a panacea against resistance. Monitoring may be helpful to find resistance hotspots and identify driving factors selecting for and spreading resistance, it will never be enough to solve the problem.

 In conclusion, the data are useful, but the text is to be improved greatly for this manuscript to become a good article.

Is the work clearly and accurately presented and does it cite the current literature?

No

If applicable, is the statistical analysis and its interpretation appropriate?

No

Are all the source data underlying the results available to ensure full reproducibility?

No source data required

Is the study design appropriate and is the work technically sound?

Partly

Are the conclusions drawn adequately supported by the results?

Partly

Are sufficient details of methods and analysis provided to allow replication by others?

Yes

Reviewer Expertise:

Antimicrobial resistance

I confirm that I have read this submission and believe that I have an appropriate level of expertise to state that I do not consider it to be of an acceptable scientific standard, for reasons outlined above.

# Reviewer report: Reviewer response for version 3

**DOI:** 10.5256/f1000research.144571.r169360

**Published:** 2023-04-28

**Authors:** Eman S. Ibrahim

**Affiliations:** 1 Department of Microbiology and Immunology, National Research Centre, Dokki, Egypt

The authors had covered all the amends required and there is no further comments to make.

Is the work clearly and accurately presented and does it cite the current literature?

Yes

If applicable, is the statistical analysis and its interpretation appropriate?

Yes

Are all the source data underlying the results available to ensure full reproducibility?

Yes

Is the study design appropriate and is the work technically sound?

Partly

Are the conclusions drawn adequately supported by the results?

Yes

Are sufficient details of methods and analysis provided to allow replication by others?

Yes

Reviewer Expertise:

Microbiology (bacteriology), Molecular biology, Nanotechnology

I confirm that I have read this submission and believe that I have an appropriate level of expertise to confirm that it is of an acceptable scientific standard.

# Reviewer report: Reviewer response for version 2

**DOI:** 10.5256/f1000research.143258.r161608

**Published:** 2023-02-17

**Authors:** Eman S. Ibrahim

**Affiliations:** 1 Department of Microbiology and Immunology, National Research Centre, Dokki, Egypt

Dear respected colleagues,

 I have read the manuscript entitled ‘Evaluation of antimicrobial susceptibility of *Escherichia coli *isolated from contaminated areas of Majengo slum in Meru County, Kenya ’ thoroughly.

 The manuscript is discussing an interesting topic; Which is the danger of antibiotic resistance concerning *E.coli *and the **linkage between antibiotic resistance and poor hygienic condition in rural areas and misuse of antibiotic treatment which in turn make it necessary to make antibiogram for choosing drug of choice before beginning treatment scheme. The work is clearly and accurately presented and cite the current literature.

 The work is technically sound but the study design is partially appropriate as the study here made screening against only five antibiotic disc which is not enough to build on it a conclusion. Can you explain why was this antibiotic chosen over others?

 Also why was genotypic identification of antibiotic resistant genes not taken?

 In the conclusions found in the abstract section you mentioned that the isolated *E.coli* is pathogenic. What is your practical support for this information as you didn't make detection for virulence genes? So it is preferable to remove the word pathogenic.

 The methods and statistical analysis and it's interpretation as well as result is well presented.

 In discussion section concerning reference 15 this paper was discussing *Salmonella isolates *how are you comparing your result with this paper? It is preferable to remove it.

 Conclusion section should include future scope of the study.

Is the work clearly and accurately presented and does it cite the current literature?

Yes

If applicable, is the statistical analysis and its interpretation appropriate?

Yes

Are all the source data underlying the results available to ensure full reproducibility?

Yes

Is the study design appropriate and is the work technically sound?

Partly

Are the conclusions drawn adequately supported by the results?

Yes

Are sufficient details of methods and analysis provided to allow replication by others?

Yes

Reviewer Expertise:

Microbiology (bacteriology), Molecular biology, Nanotechnology

I confirm that I have read this submission and believe that I have an appropriate level of expertise to confirm that it is of an acceptable scientific standard, however I have significant reservations, as outlined above.

# Response

**Published:** 2023-03-10

**Authors:** jared ombuya

**Affiliations:** meru university, Kenya

The reason why I concluded that the organism is pathogenic is that I did serotyping of E coli strains and one strain was positive for the test

 The reason for using the 5 antibiotics is that those are the commonly prescribed drugs in the health centers in the study area

 Genotyping was not done as that could have quite expensive for me but that's the work to be done in my next level of study

 The reference included for salmonella was mistakenly included hence will not appear in revised version

 Conclusion will be amended accordingly as well in the revised version
